# Computer-aided diagnosis system for bone scintigrams from Japanese patients: importance of training database

**DOI:** 10.1007/s12149-012-0620-5

**Published:** 2012-06-24

**Authors:** Hiroyuki Horikoshi, Akihiro Kikuchi, Masahisa Onoguchi, Karl Sjöstrand, Lars Edenbrandt

**Affiliations:** 1 Department of Diagnostic Radiology, Gunma Prefectural Cancer Center, Takabayashi Nishimachi 617-1, Ota, Gunma 373-0828 Japan; 2 Division of Health Sciences, Graduate School of Medical Science, School of Health Sciences, College of Medical, Pharmaceutical and Health Sciences, Kanazawa University, 5-11-80 Kodatuno, Kanazawa, Ishikawa 920-0942 Japan; 3 Informatics and Mathematical Modeling, Technical University of Denmark, Asmussens Alle, building 305, Lyngby, 2800 Copenhagen, Denmark; 4 Department of Molecular and Clinical Medicine, Sahlgrenska Academy, Gothenburg University, 405 30 Gothenburg, Sweden

**Keywords:** Computer-aided diagnosis, Bone scintigram, Bone metastases, Artificial neural networks

## Abstract

**Aim:**

Computer-aided diagnosis (CAD) software for bone scintigrams have recently been introduced as a clinical quality assurance tool. The purpose of this study was to compare the diagnostic accuracy of two CAD systems, one based on a European and one on a Japanese training database, in a group of bone scans from Japanese patients.

**Method:**

The two CAD software are trained to interpret bone scans using training databases consisting of bone scans with the desired interpretation, metastatic disease or not. One software was trained using 795 bone scans from European patients and the other with 904 bone scans from Japanese patients. The two CAD softwares were evaluated using the same group of 257 Japanese patients, who underwent bone scintigraphy because of suspected metastases of malignant tumors in 2009. The final diagnostic results made by clinicians were used as gold standard.

**Results:**

The Japanese CAD software showed a higher specificity and accuracy compared to the European CAD software [81 vs. 57 % (*p* < 0.05) and 82 vs. 61 % (*p* < 0.05), respectively]. The sensitivity was 90 % for the Japanese CAD software and 83 % for the European CAD software (n.s).

**Conclusion:**

The CAD software trained with a Japanese database showed significantly higher performance than the corresponding CAD software trained with a European database for the analysis of bone scans from Japanese patients. These results could at least partly be caused by the physical differences between Japanese and European patients resulting in less influence of attenuation in Japanese patients and possible different judgement of count intensities of hot spots.

## Introduction

Patients with prostate or breast cancer often have bone metastases, and the bone metastases of the primary carcinomas may greatly affect the management and prognosis of the patient, so the diagnosis of metastatic disease is important [1–3]. The interpretation of bone scans and other diagnostic images are generally made visually as a pattern recognition task where for example the accumulation of a radiopharmaceutical is assessed. This type of task is subjective and largely dependent on the experience of the interpreter. Computer-aided diagnosis (CAD) software has been introduced in practical use as an objective approach to interpret diagnostic images. The computer interpretations can be used as a second opinion. CAD software has been developed for mammography, colonography and myocardial perfusion scintigrams [4–7]. In the USA, CAD software has been used for a part of clinical routine for detecting breast cancers by mammography for several years [8]. Lindahl et al. [9] reported that physicians improved their diagnostic capability significantly by the use of CAD software in for the analysis of myocardial perfusion scintigrams.

CAD software for the interpretation of bone scans have also been developed recently [10–14]. Sadik et al. [12] reported that even an experienced physician obtained benefits for diagnosis through the CAD software and that differences in interpretations between observers decreased. This bone scan CAD software was trained to interpret bone scans using training databases consisting of bone scans from European patients with the desired image interpretation, metastatic disease or not. It is well known that American and European standard values are not appropriate to use in myocardial scintigrams for Japanese patients [15]. An assumption could be that it is of value to use a database consisting of bone scans from Japanese patients for training of CAD software for Japanese patients. The purpose of this study was to compare the diagnostic accuracy of two CAD systems, one based on a European and one on a Japanese training database, in a group of bone scans from Japanese patients.

## Materials and methods

### Patients

The evaluation group of this study comprised 257 patients (males 113, females 144; age 63± years), who had underwent bone scintigrams for detecting bone metastatic diseases in 2009. The primary cancers included 125 breast cancers, 64 prostatic cancers, 34 lung cancers, 9 bladder cancers, 4 gastric cancers, 5 renal cancers, 3 esophagus cancers, 2 testicular cancers, 2 ureteral cancers, 2 rectal cancers, 1 hepatoma, 1 endometrial cancer, 1 multiple myeloma, 1 bile duct cancer, 1 oropharyngeal cancer, 1 ovarian cancer and 1 lymphoma.

The final clinical assessment of each patient, made by an experienced physician, was used as the gold standard classification for the presence or absence of bone metastases. These assessments were based on increase and decrease of the high accumulation areas, changes in intensity and sizes by follow-up bone scans and other diagnostic imaging modalities such as MRI, CT, radiographic images or blood data.

### CAD software

The CAD software interprets the bone scans automatically regarding metastatic disease. The method for interpretation consists of image analysis and artificial neural networks (ANNs). The CAD software is fed with the anterior and posterior images in digital format. The first steps are image segmentation, detection and characterization of high accumulation areas. The segmentation of the skeleton is performed using a normal atlas comprising 14 anatomical segments. The skeleton is normalized and high accumulation areas are detected in an iterative process. A characterization of high accumulation area is performed and the resulting features include intensity, shape and localization. The resulting image features are used as input to ANNs classifying high accumulation areas. A second set of neural networks is used to provide the ANN value on the basis of the results of the first set of networks. The neural networks are trained using databases of classified examples of bone scans. Two different European and Japanese training databases were used. After the training process, all bone scans of the evaluation group were processed with the two CAD softwares.

#### European training database

The European database consists of data of the cases which underwent examination at Sahlgrenska University Hospital in Gothenburg, Sweden from January 1999 to June 2002. The final number of cases was 795 including 514 males (70.2 ± 9.7 years old, age range 27–91 years) and 281 females (58.9 ± 13.1 years old, age range 25–92 years). Cases with technical problems during acquisition were excluded.

#### Japanese training database

The Japanese database is based on cases with diagnoses established at Gunma Prefectural Cancer Center in the years of 2007 and 2008. The final number of cases was 904 including 457 males (68.4 ± 10.4 years old, age range 20–91 years) and 447 females (58.5 ± 12.5 years old, age range 26–91 years). For diagnosis of metastasis, two expert radiologists made final diagnosis based on blood data, MRI or CT, and clinical course of 1 year or more. The cases included lung cancer, breast cancer, prostatic cancer, colorectal cancer and degenerative diseases. Cases of the ileal conduit were excluded.

**Fig. 1:**
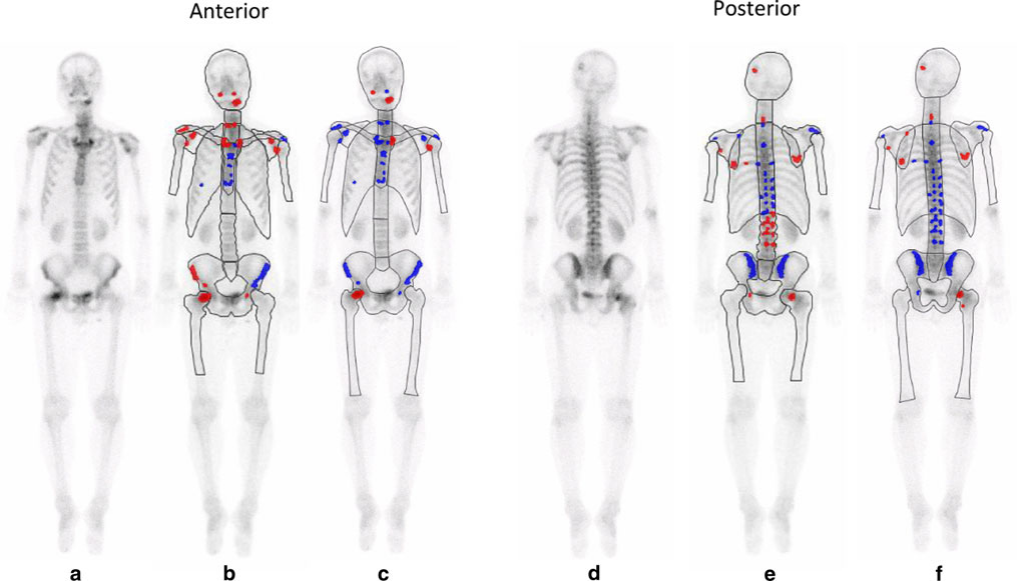
A 55-year-old man with lung cancer. Increased radiotracer uptake can mainly be seen in cranial bone, mandible, cervical spine, sternum, and right femur on whole-body scan (**a**, **d**). CAD software with European database (**b**, **e**) classifies as this patient as having no metastases (ANN 0.29, grade 2). However, CAD software with Japanese database (**c**, **f**) correctly classifies as having metastases (ANN 0.87, grade 4). CT imaging shows osteolytic bone metastasis corresponding to increased radiotracer uptake of cranial bone and right femur (not shown)

Aiming at bone whole-body scan images (anterior and posterior images), a result is displayed through roughly two analysis flows comprising image analysis and ANN analysis as shown in Fig. 1. The analysis software BONENAVI (FUJIFILM RI Pharma Co., Ltd.) used in this study performs from analysis after data input to output almost automatically, and is excellent in convenience or repeatability.

### Image evaluation using CAD software

A cutoff ANN value of 0.5 was chosen to provide the CAD software with the binary classification of ‘‘bone metastases’’ or ‘‘no bone metastases.’’ All cases with ANN values were assigned following grades.

### Grade 1 (ANN 0–0.25)

Some cases without findings of suspected bone metastases had high accumulation areas by bone scintigrams. For high accumulation areas, images showed normal accumulation patterns or degenerative change or bone fracture. Any patterns indicating bone metastases were also not observed by other modalities.

### Grade 2 (ANN 0.26–0.50)

Cases in which bone metastasis may not be excluded securely by bone scintigrams. Although changes such as increase of intensity or expansion of the range were not observed in high accumulation areas at follow-up, changes due to bone fracture or degenerative diseases were observed by other modalities. It was determined that bone metastases were less suspicious by clinical judgment.

### Grade 3 (ANN 0.51–0.75)

Cases with bone metastases suspected by bone scintigrams. Changes such as increase of intensity or expansion of the range were observed in high accumulation areas at follow-up, or findings suspecting bone metastases were obtained by other modalities and bone metastases were clinically suspected.

### Grade 4 (ANN: 0.76–1.00)

Cases with bone metastases highly suspected by bone scintigrams. Typical bone metastatic patterns were observed in high accumulation areas. Typical bone metastatic patterns were observed by other modalities as well.

## Bone scintigrams

Whole-body bone scan images, anterior and posterior views, were acquired 3–5 h after an intravenous injection of 740 MBq Technetium-99 m methylene diphosphonate (FUJIFILM RI Pharma Co., Ltd., Japan). A dual-detector gamma-camera system equipped with a low energy high resolution parallel hole collimators was used Siemens ECAM. The acquisition conditions of the whole-body scan were set as follows: scan speed 15 cm/min, matrix size 256 × 1024, energy window of energy peak 140 keV and window width 15 %.

## Statistical analysis method

Cases classified as Grade 1 or Grade 2 were regarded as having no metastases and Grade 3 or Grade 4 had metastases. Differences in sensitivity, specificity and accuracy between the two CAD were tested using a McNemar test.

## Results

The final clinical assessment showed 42 cases with bone metastases and 215 cases without bone metastases. The number of true positive classifications was 38 cases for the Japanese CAD software and 35 cases for the European CAD software and this difference was not statistically significant. The number of true negative classifications was 174 cases for the Japanese CAD software and 122 cases for the European CAD software. The classifications of the CAD software for the Japanese and European databases are presented in Table 1. The sensitivity, specificity, and accuracy for the Japanese and European CAD software are presented in Table 2. The Japanese CAD software showed a higher specificity and accuracy compared to the European CAD software [81 vs. 57 % (*p* < 0.05) and 82 vs. 61 % (*p* < 0.05), respectively]. The sensitivity was 90 % for the Japanese CAD software and 83 % for the European CAD software (n.s).

**Table 1:** Classifications of the CAD software for the Japanese and European databases

Grade	1	2	3	4	Total
Japanese database CAD					
Bone metastases	4	0	3	35	42
No bone metastases	163	11	7	34	215
European database CAD					
Bone metastases	3	4	2	33	42
No bone metastases	67	55	30	63	215

*n* numbers

**Table 2:** Comparison of CAD software according to Japanese and European databases

	Japan database CAD (%)	European database CAD (%)
Sensitivity	90	83
Specificity	81*	57*
Accuracy	82*	61*

* *p* < 0.05

## Discussion

Usefulness of the CAD system for bone scintigrams using the ANN technology used in this study was reported by the Sadik et al. [10–12]. A high disease detection rate and sensitivity of almost 90 % were indicated in the report [12]. In the analysis software used in this study, skeletal segmentation, detection of a high accumulation area, characterization and an ANN algorithm were used.

In this study, difference in the analysis results due to the difference in the races of the databases was compared, showing that a better diagnostic result for Japanese patients could be obtained by use of the Japanese database (Fig. 1). As long as seeing the reference analysis result, the dose and collection conditions of the radiopharmaceutical of each database were almost equivalent. The system resolution was 8.1 mm for Maxxus and 7.4 mm for ECAM, which were comparable. In analysis, the ANN value in the high accumulation area has greatly accounted for the final result, and the distribution and intensity may be affected by difference in the absorption of radiation due to difference in the physical constitution.

In judgment using the ANN value, the count intensity of the high accumulation area is a key factor. For the count intensity, influence of internal absorption depending on the physical constitution becomes a major variation factor if the dose and the collection conditions are the same. Especially absorption in the trunk area tends to be different, and this area varies significantly by races. The high accumulation area in the trunk area is critical in characterization by the ANN [13], and since Japanese are small in stature on the average compared with Europeans, influence of internal absorption is less than in Europeans, and the count intensity in the high accumulation area may tend to be higher consequently. Using the European database tends to result in false positive, while sensitivity and specificity may have been improved using the Japanese database including the characteristics of the Japanese physical constitution.

## Conclusion

It was suggested that the precision, especially the specificity and the accuracy of the CAD system could be improved significantly by creating a Japanese database when analyzing the Japanese data.
